# Injury to the Penis during Coition

**Published:** 1890-06

**Authors:** Alexander A. Nevsky

**Affiliations:** Gorokhovetz, Russia


### INJURY TO THE PENIS DURING COITION.

BY DR. ALEXANDER A. NEVSKY (Gorokhovetz, Russia).

A healthy and strong gentleman, æt. 25 years, while performing coitus with his wife, suddenly felt an intense acute ("as if cutting") pain about the penis. On immediately withdrawing the organ, he discovered a profusely bleeding laceration. On examining the highly excited patient about half an hour later (during which time the gentleman had lost about half-a-cupful of blood in spite of a continuous application of cold, pressure, etc.) the author found a widely gaping, irregular, deep, lacerated wound with everted and tumefied edges, measuring in length more than an inch, and running transversely along the posterior or lower aspect of the member about 1 inch from the edge of the foreskin. The latter proved to be unusually elongated and tight, its orifice very narrow, so that the glands could be exposed only to a very slight extent. The wound was at once washed out with a 20 per cent. boracic acid lotion and closed with silk sutures, after which the hemorrhage ceased. In five or six days, the lesion healed *per primam.* As to the mechanism of the injury, Dr. Nevsky suggests that during the sexual act the patient's long and phymotic prepuce formed a terminal fold looking upward; which led to an extreme stretching of the skin on the lower surface of the organ, the tissues giving way at the point of a maximal distention under some violent pressure of the glands. Pointing out that traumatic injuries to the penis during coitus represent a very rare occu r rence, the author says that he has been able to find only two instances of the kind in recent international literature, both being communicated by American practitioners. They are Dr. Veazie's case of complete fracture of the penis (*New Orleans Medical Journal, 1884*) and Dr. Egerton Davis' (Philadelphia) of the member in the vagina (*Deutsche Medieinische Zeitung*, 1885, August 6.) [In the *Lancet*, February 18, 1888, p. 321, Dr. Hulke, of London, relates a very interesting case of sprained penis, with a consecutive, long-continued priapism caused by inflammation of the lacerated left crus. The patient, an artisan, æt. 34 years, was heavily intoxicated during the intercourse with his wife.—REPORTER.]—*Vracth*, No. 48, 1889, p. 1058.—*Annals of Surgery.*

